# Differential diagnosis of benign and malignant breast masses using diffusion-weighted magnetic resonance imaging

**DOI:** 10.1186/s12957-014-0431-3

**Published:** 2015-02-07

**Authors:** Qinghua Min, Kangwei Shao, Lulan Zhai, Wei Liu, Caisong Zhu, Lixin Yuan, Jun Yang

**Affiliations:** Department of Radiology, Shanghai Tongren Hospital, No. 1111 Xianxia Road, Changning District, Shanghai, 200336 PR China

**Keywords:** breast lesions, *b* value, differential diagnosis, diffusion-weighted imaging, magnetic resonance imaging

## Abstract

**Background:**

Diffusion-weighted magnetic resonance imaging (DW-MRI) is different from conventional diagnostic methods and has the potential to delineate the microscopic anatomy of a target tissue or organ. The purpose of our study was to evaluate the value of DW-MRI in the diagnosis of benign and malignant breast masses, which would help the clinical surgeon to decide the scope and pattern of operation.

**Methods:**

A total of 52 female patients with palpable solid breast masses received breast MRI scans using routine sequences, dynamic contrast-enhanced imaging, and diffusion-weighted echo-planar imaging at *b* values of 400, 600, and 800 s/mm^2^, respectively. Two regions of interest (ROIs) were plotted, with a smaller ROI for the highest signal and a larger ROI for the overall lesion. Apparent diffusion coefficient (ADC) values were calculated at three different *b* values for all detectable lesions and from two different ROIs. The sensitivity, specificity, positive predictive value, and positive likelihood ratio of DW-MRI were determined for comparison with histological results.

**Results:**

A total of 49 (49/52, 94.2%) lesions were detected using DW-MRI, including 20 benign lesions (two lesions detected in the same patient) and 29 malignant lesions. Benign lesion had a higher mean ADC value than their malignant counterparts, regardless of *b* value. According to the receiver operating characteristic (ROC) curve, the smaller-range ROI was more effective in differentiation between benign and malignant lesions. The area under the ROC curve was the largest at a *b* value of 800 s/mm^2^. With a threshold ADC value at 1.23 × 10^−3^ mm^2^/s, DW-MRI achieved a sensitivity of 82.8%, specificity of 90.0%, positive predictive value of 92.3%, and positive likelihood ratio of 8.3 for differentiating benign and malignant lesions.

**Conclusions:**

DW-MRI is an accurate diagnostic tool for differentiation between benign and malignant breast lesions, with an optimal *b* value of 800 s/mm^2^. A smaller-range ROI focusing on the highest signal has a better differential value.

## Background

Breast cancer is a common malignancy and cause of cancer death [1]. Despite improvements in the detection of breast cancer with the widespread application of mammography and ultrasonography, differentiation between benign and malignant breast lesions remains a difficult diagnostic problem [2]. Magnetic resonance imaging (MRI) is an established supplementary technique to mammography and ultrasonography for the evaluation of suspicious breast lesions and primarily advantageous in its higher sensitivity over both mammography and ultrasonography [3,4].

Diffusion-weighted MRI (DW-MRI) is an advanced MRI technique, which emerged in the mid-1980s and allows the mapping of *in-vivo* water diffusion processes in a non-invasive manner; it can delineate the microscopic anatomy of a target tissue or organ [5]. It has a higher sensitivity and specificity in detecting suspicious breast disease at a minimum size of 1 cm than regular MRI [5]. It can provide digital biomarker measurements of tissue properties that are highly relevant to the assessment of tumor progression or responses [6,7]. Diffusion-weighted MRI produces images that are sensitive to water displacement at the diffusion scale and quantifies such diffusion according to an index that reflects the apparent freedom of diffusion, called the apparent diffusion coefficient (ADC). It has been reported to produce higher detection rates than conventional methods [6,8] and can be easily adopted as an adjunct to standard clinical imaging protocols [9,10]. However, the reported sensitivity and specificity of DW-MRI among previous reports is varied, possibly owing to the variability of MRI equipment and scanning parameters, such as definition of the *b* value (the strength of the magnetic diffusion gradient) and region of interest (ROI) [5,11].

The primary objective of this study was to evaluate the role of DW-MRI in the diagnosis of benign and malignant breast masses. Moreover, we also attempted to optimize the *b* value, to improve the differential capability of DW-MRI between benign and malignant breast masses.

## Methods

### Patients

This study included 52 women (age range: 20 to 86 years) who underwent breast DW-MRI between March 2008 and March 2010 at Tong Ren Hospital Affiliated to Shanghai Jiao Tong University school of medicine (Shanghai, PR China) because of clinical abnormality or suspicious findings on breast ultrasonography. The sources of specimens for histological examination were surgical excision (48 cases) and core needle biopsy (4 cases). A total of 31 cases were detected in the left breast, and 21 were detected in the right breast. All patients had DW-MRI prior to the biopsy or surgical procedure. Patients under neoadjuvant chemotherapy, with history of breast biopsy within 1 month, without a detectable lesion on MRI corresponding to a clinically or mammographically defined lesion, and without histopathologic confirmation of the lesion were excluded from the study. This retrospective study was approved by the institutional review board and ethics committee of Tong Ren Hospital Affiliated to Shanghai Jiao Tong University, Shanghai, China. Written informed consent was obtained from all patients before MRI.

### MRI image acquisition

MRI was performed using a 1.5 T MR system (Sigma Excite II; GE Medical System, Milwaukee, WI, USA) and a dedicated phased-array bilateral breast coil, with the patient lying prone and the breast in a holder to reduce motion [12]. Prior to diffusion weighting, fast recovery fast spin echo was performed in the axial plane, three-dimensional fast spoiled gradient recalled acquisition in the steady state (3DFSPGR) with Spec IR for fat saturation in the sagittal plane was performed before and after administration of gadopentetate dimeglumine (0.2 mmol/kg). Subtraction images were produced with 3DFSPGR for identification of enhancement. Two-dimensional fast spoiled gradient recalled acquisition in the steady state in the axial plane was performed after enhancement. Imaging parameters were as follows: T1 sagittal plane, repetition time (TR)/echo time (TE) = 480/10.4 ms, number of excitations (NEX) = 2, slice thickness = 5 mm, and field of view (FOV) = 220 mm; T2 sagittal plane, TR/TE = 3,500/85 ms, NEX = 3, slice thickness = 5 mm, and FOV = 220 mm; axial plane, TR/TE = 4,000/85 ms, NEX = 3, slice thickness = 4 mm, FOV = 160 mm. One pre-contrast and six continuous dynamic contrast-enhanced spoiled gradient echo (SPGR) three-dimensional acquisitions were performed using a rapid bolus injection of 0.2 mmol/kg gadolinium-chelated diethylenetriaminepentaacetic acid (Magnevist, Schering, Berlin, Germany), at a TR/TE of 6.6/2.2 ms, flip angle of 15°, and 3-mm slice thickness for the sagittal plane. Diffusion-weighted axial images with single-shot echo-planar imaging (TR/TE = 9,355 ms/78.5 ms, matrix = 128 × 128, NEX = 4, FOV = 280 mm, slice thickness = 5 mm, and slice gap = 0 mm) were captured at *b* values of 400, 600 and 800 s/mm^2^, respectively. Double axial post-contrast SPGR T1-weighted imaging was performed to complete the MRI scan.

### Image analysis

**Figure 1:**
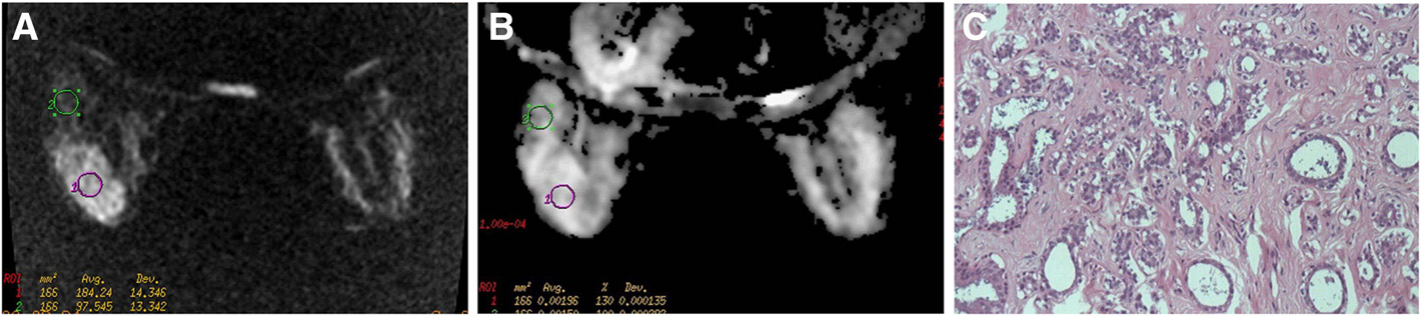
**A representative case of benign breast lesion: sclerosing mastopathy (in a 53-year-old patient). (A)** Axial diffusion-weighted MRI map. The lesion shows as an oval mass with a circumscribed margin. **(B)** Axial apparent diffusion coefficient (ADC) imaging (*b* value is 800 s/mm^2^): The ADC of the lesion is 1.96 × 10^−3^ mm^2^/s. **(C)** Micrograph with H & E stain (original magnification, 200×). The lobule of the mammary gland displays adenomatoid hyperplasia and extensive fibrous stroma and demonstrates pseudoinvasion.

**Figure 2:**
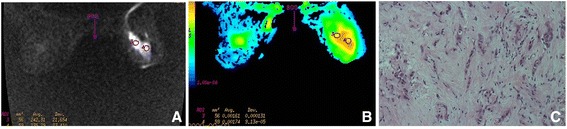
**A case of malignant breast lesion: invasive lobular carcinoma (in a 71-year-old patient). (A)** Axial diffusion-weighted MRI map with *b* value of 800 s/mm^2^, showing scattered foci with high signal intensity. **(B)** ADC map. The absolute ADC is 1.61 × 10^−3^ mm^2^/s, which is consistent with benign lesion, owing to the ADC cut-off value of 1.23 × 10^−3^ mm^2^/s. **(C)** Micrograph with H & E stain (original magnification, 400×). The extracellular space that small cancer cells form is extensive.

Evaluation of images was performed in consensus by two MRI-accredited radiologists with more than 5 years’ experience each. They were assigned to identify and locate the breast disease shown on DW-MRI images by referring to the dynamic-enhanced imaging (Figure 1A, Figure 2A). The DW-MRI quality was assessed with respect to fat suppression, blurring and other artifacts, and signal intensity [13,14]. Each patient had three ADC maps created at *b* values of 400, 600 and 800 s/mm^2^, respectively. Each ADC was mapped in a colour or gray-scale format (Figure 1B, Figure 2B). To resolve disagreement between observers, a third radiologist (with 5 years of experience in breast imaging and 20 years of experience in MRI) assessed all involved items. Regions of interest were drawn in the breast parenchyma in the center of the contralateral breast, avoiding contamination by fatty tissue. The ROIs were then copied and pasted onto the corresponding ADC map for quantitative analysis. For each ROI, we extracted the mean ADC and the ROI area [6]. The ADC values were automatically calculated using the Functool 2 software of the GE ADW 4.1 workstation by placing two separate ROIs on the target lesions and according to the equation:

$$ \mathrm{A}\mathrm{D}\mathrm{C}=1\mathrm{n}\left(\mathrm{S}\mathrm{i}/\mathrm{S}0\right)/\left(\mathrm{bi}-\mathrm{b}0\right) $$

where *S*_0_ is the first acquired image at *b* = 0 s/mm^2^, *S*_*i*_ are the corresponding images at *b* = 400, 600, and 800 s/mm^2^, *b*_0_ is the *b* value, and *b*_*i*_ is the diffusion gradient value.

The measuring area was kept constant among images with three *b* values. Use of a higher *b* value reduced the contribution of perfusion effects in ADC measurements, according to the equation. Small differences between ADC values were observed at lower *b* values, with larger differences at higher *b* values. The lower ADC in malignancies was primarily attributed to a higher cell density, causing increased restriction of the extracellular matrix; there was an increased fraction of signal coming from intracellular water. The regions selected as clearly identifiable had high signal intensity on diffusion-weighted MRI, yet we avoided regions of high signal intensity on T2-weighted images, to exclude the T2 shine-through effect. A smaller-scale ROI was plotted on the highest-signal area of the lesion at a minimum size of 26 mm^2^, while a larger-scale ROI was also plotted, to outline the full-scale disease. An oversized ROI (too large to outline at full-scale) was divided into multiple smaller-scale ROIs to produce the mean ADC. Diagnostic sensitivity, specificity, positive predictive value, and positive likelihood ratio of DW-MRI were produced for comparison with histological results (Figure 1C, Figure 2C).

#### Statistical analysis

The SPSS software package version 16.0 (SPSS Inc., Chicago, IL, USA) was used for statistical analysis. Continuous data were expressed as mean ± standard deviation and compared using the independent sample Mann-Whitney *U* test or the paired Student-*t* test, and nonparametric data were analyzed using the related-samples Friedman test. The cut-off values differentiating benign and malignant breast diseases were defined using receiver operating characteristic (ROC) curves. A two-tailed *P* < 0.05 was considered statistically significant.

## Results

### Detectability on DW-MRI

All 52 patients enrolled in this study successfully underwent DW-MRI for their suspicious breast findings and had a histopathologic reference standard test for their index lesion. Four of 52 lesions were not detected on DW-MRI (detectability rate: 49/52, 94.2%), and all of the four lesions were confirmed to be diffuse cystic mastopathy. There were 29 malignant lesions in 29 patients, including 26 invasive ductal carcinomas in 26 patients and 3 invasive lobular carcinomas in 3 patients. There were 20 benign lesions in 23 patients, including one fibrocystic changes in one patient, four instances of plasma cell mastitis in three patients (two lesions were detected in one of the three patient), one intraductal papilloma in one patient, ten fibroadenoma in ten patients, two instances of mastopathy in six patients, one sclerosing mastopathy in one patient, and one abscess in one patient (Table 1). There was no significant difference in the detection rate of DW-MRI among the three *b* values (*P* > 0.05).

**Table 1:** **Detection rate of diffusion-weighted MRI by histology**

**Histology**	**Number of patients**	**Number of cases of MRI-detectable disease**	**Size (cm)**
Benign disease (*n* = 23)			
Fibrocystic changes	1	1	1.3
Plasma cell mastitis	3	4	4.6 to 5.2
Intraductal papilloma	1	1	1.4
Fibroadenoma	10	10	0.6 to 4.8
Mastopathy	6	2	1.6, 3.7
Sclerosing mastopathy	1	1	5.0
Abscess	1	1	2.7
Malignant disease (*n* = 29)			
Invasive ductal carcinoma	26	26	1.0 to 3.6
Invasive lobular carcinoma	3	3	1.2 to 3.5
Total	52	49	

MRI, magnetic resonance imaging.

### Imaging quality and signal analysis

Diffusion-weighted MRI exhibited a superior readability with a good fat suppression and signal intensity in all patients, except for one patient with some margin artifacts. Benign lesions showed a similar or slightly lower signal intensity than parenchyma in ADC maps, while the great majority of malignant lesions showed a significantly lower signal intensity than parenchyma. According to non-mass-like enhancement on dynamic-enhanced imaging, the *b* value of 800 s/mm^2^ could significantly improve the imaging of 11 lesions. It also exhibited a significantly higher signal contrast between the lesions and the parenchyma than *b* values of 400 and 600 s/mm^2^. No significant differences were observed in mass-like enhancement among the three *b* values.

### Analysis of ADCs

**Figure 3:**
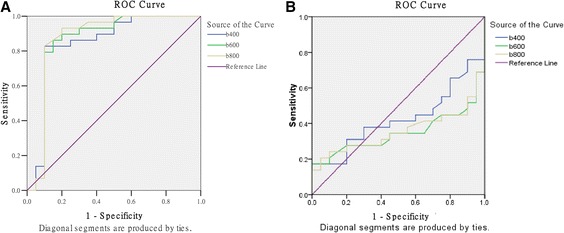
**Comparison of receiver operating characteristic (ROC) curves for three** ***b*** **values. (A)** Smaller-range region of interest for different *b* values. **(B)** Larger range of region interest for different *b* values.

The mean ADC for malignant masses was lower than the mean ADC for benign lesions among three *b* values (all *P* < 0.05; Table 2). The effect of the ROI scale on ADC is shown in Table 3. The smaller- and larger-scale ROIs exhibited statistically significant differences in ADC. The ROC analysis showed that the smaller-scale ROI was more effective in differentiating between benign and malignant diseases (Figure 3). For *b* values of 400, 600 and 800 s/mm^2^, the ROC area under the curve was 0.847, 0.861, and 0.875, respectively. The area was largest when the *b* value was 800 s/mm^2^ (95% confidence interval 0.746 to 1.004), indicating that a *b* value of 800 s/mm^2^ may be optimal for differentiation between malignant and benign breast lesions.

**Table 2:** **ADC values of MRI-detectable benign/malignant breast diseases (n = 49)**

***b*** **value (s/mm** ^**2**^ **)**	**Breast disease**	**Mean ADC (×10** ^**−3**^ **mm** ^**2**^ **/s)**	**Mean square**	**Standard deviation**	**95% confidence interval**	***P***
400	Benign	1.85	0.19	0.44	0.80 to 2.62	<0.001
	Malignant	1.36	0.07	0.27	1.01 to 1.97	
600	Benign	1.79	0.28	0.53	0.65 to 2.90	<0.001
	Malignant	1.21	0.05	0.23	0.78 to1.79	
800	Benign	1.66	021	0.46	0.64 to 2.47	<0.001
	Malignant	1.11	0.04	0.19	0.78 to 1.69	

ADC, apparent diffusion coefficient; MRI, magnetic resonance imaging.

**Table 3:** **Effect of** ***b*** **value on ADC value difference between large- and small-scale regions of interest for MRI-detectable breast diseases (** ***n*** **= 49)**

***b*** **value (s/mm** ^**2**^ **)**	**Mean ADC difference**	**Standard deviation**	**95% confidence interval**	***P*** **-value**
400	0.07	0.15	0.03 to 0.12	0.001
600	0.07	0.13	0.03 to 0.11	0.001
800	0.05	0.12	0.01 to 0.08	0.008

ADC, apparent diffusion coefficient; MRI, magnetic resonance imaging.

### Sensitivity, specificity, positive predictive value, and positive likelihood ratio

With a predefined ADC cut-off value at 1.23 × 10^−3^ mm^2^/s, DW-MRI gave a sensitivity of 82.8%, specificity of 90.0%, positive predictive value of 92.3%, and positive likelihood ratio of 8.28, in differentiation between benign and malignant diseases (Table 4). Three invasive ductal carcinoma cases and two invasive lobular carcinoma cases among 29 malignant cases were misdiagnosed as benign; 1 adenosis case and one abscess case out of 23 benign cases were mistakenly diagnosed as malignant.

**Table 4:** **Differentiation capability of ADC value for MRI-detectable benign and malignant breast diseases (** ***n*** **= 49)**

***b*** **value (s/mm** ^**2**^ **)**	**Mean ADC of benign disease (×10** ^**−3**^ **mm** ^**2**^ **/s)**	**Mean ADC of malignant disease (×10** ^**−3**^ **mm** ^**2**^ **/s)**	**Sensitivity**	**Specificity**	**Positive predictive value**	**Positive likelihood ratio**
400	1.85 ± 0.86	1.36 ± 0.53	82.8%	90.0%	92.3%	8.28
600	1.79 ± 1.03	1.21 ± 0.45	86.2%	85.0%	86.2%	5.74
800	1.66 ± 0.90	1.11 ± 0.37	82.8%	90.0%	92.3%	8.28

ADC, apparent diffusion coefficient; MRI, magnetic resonance imaging.

## Discussion

This study assessed the role of DW-MRI in the diagnosis of benign and malignant breast masses, with the goals of providing guidance for clinical diagnosis. The results revealed that DW-MRI had an overall detection rate of 94.2% for benign and malignant breast diseases, relatively higher than that (86.2%) reported by Park *et al.* [15]. Moreover, the performance of DW-MRI was affected by *b* value, ADC threshold, and ROI scale.

Visualization of DW-MRI depends on water molecule diffusion, which will be influenced by histological structure; in other words, the signal intensity will imply the histological structure. Diffusion is quantified by measuring what is known as the ADC value in square millimeters per second, which defines the average area covered by a molecule per unit time. The ADC value can be calculated by assessing the signal attenuation that occurs at diffusion-weighted imaging performed at different *b* values [16]. Many factors can influence ADCs, including imaging parameters and pathophysiologic features of the lesions [16]. The motion of water molecules is subject to more restriction in tissues with a high cellular density or enriched with lipophilic barrier membranes. Malignant tumors usually have a high cellular density, contractible extracellular space, and great absorption of biomass molecules, and consequently restrict water molecule diffusion.

Diffusion-weighted MRI has been accepted as a powerful adjuvant to conventional MRI for reducing false positivity in breast cancer screening. Prior investigators (Table 5) have evaluated the role of MRI in breast masses performed with 1.5 T systems and reported that ADCs have the potential to help differentiate benign from malignant lesions. Our results showed that DW-MRI had an overall detection rate of 94.2% for benign and malignant breast diseases, relatively higher than previously reported [15]. It also demonstrated that the detective and imaging quality of DW-MRI remained for *b* values of 400, 600 and 800 s/mm^2^; however, a *b* value of 800 s/mm^2^ produced a higher-contrast resolution between the non-mass-like enhancement tissue and the normal parenchyma than did *b* values of 400 and 600 s/mm^2^. Moreover, consistent with several previous studies [4,17,18], the current study revealed that the ADC value of malignant lesions was significantly lower than that of benign lesions. The ADC exhibited a gradual increase from the innermost layer to the outermost layer at the tumor margin. In addition, accurate positioning of the ROI is essential for an accurate measure of ADC [19]. Our results showed that the encompassed smaller-scale ROI had a constantly smaller ADC and a better differential capability than the larger-scale counterpart. These were consistent with previous studies [14–16,20–22]. Therefore, the smaller-scale ROI in DW-MRI was recommended for differentiating benign and malignant breast diseases.

**Table 5:** **ADC threshold and sensitivity/specificity of breast DW-MRI**

**Reference**	**ADC threshold (** **×10** ^**−3**^ ** mm** ^**2**^ **/s** **)**	**Sensitivity (%)**	**Specificity (%)**
Lalitha *et al* [23]	1.3 to 1.5/0.85 to 1.1(for benign/malignant disease)	97.2	100.0
Zhang *et al.* [12]	1.24/1.2 (*b* = 500/1,000 s/mm^2^)	93.0/96.0	100.0/97.0
Luo *et al.* [24]	1.22	88.9	87.9
Reiko *et al.* [16]	1.6	95.0	46.0
Marini *et al.* [25]	1.1	80.0	81.0
Guo *et al.* [26]	1.3	93.0	88.0
Woodhams *et al.* [27]	1.6	93.0	46.0
Ruboseva *et al.* [19]	1.13	86.0	86.0
Hatakenaka *et al.* [28]	1.48	83.9	81.3
Pereira *et al.* [4]	1.21	92.3	92.3
Our study	1.23	82.8	90.0

ADC, apparent diffusion coefficient; DW-MRI, diffusion-weighted magnetic resonance imaging.

There were some limitations to this retrospective study. Firstly, although the total number of lesions evaluated was relatively large, there were a relatively small number of benign diseases for each type. Secondly, with the described technique, these processes could potentially be automated for evaluation of the entire breast rather than a focal lesion; however, ADC determination and ROI selection were conducted by a single blinded reader. Finally, since all of the MRI examinations were performed using a single MRI platform, the results might be specific to certain pulse sequences. Despite these limitations, DW-MRI of the breast provides additional information for characterizing focal breast lesions in a fast and easy way. In this series, we obtained sensitivity and specificity as high as 82.8% and 90.0%, respectively. Nevertheless, further studies with larger populations are needed to confirm the value of DW-MRI in the evaluation of breast lesions.

## Conclusions

In summary, the study revealed that DW-MRI had a good performance in differentiating both benign and malignant diseases at breast. With a *b* value of 800 s/mm^2^, an ADC threshold at 1.23 × 10^−3^ mm^2^/s and an encompassed smaller-scale ROI, breast DW-MRI exhibited a moderately high sensitivity and specificity in differentiation between benign and malignant lesions. Larger-scale randomized controlled studies are required to validate the superiority of DW-MRI.

## Abbreviations

3DFSPGR: three-dimensional fast spoiled gradient recalled acquisition in the steady state
ADC: apparent diffusion coefficient
DW-MRI: diffusion-weighted MRI
FOV: field of view
H & E stain: MRI, magnetic resonance imaging
NEX: number of excitations
ROC: receiver operating characteristic
ROI: region of interest
SPGR: spoiled gradient echo
TE: echo time
TR: repetition time
